# Traditional and Advanced Cell Cultures in Hematopoietic Stem Cell Studies

**DOI:** 10.3390/cells8121628

**Published:** 2019-12-12

**Authors:** Antonio Carlos Ribeiro-Filho, Débora Levy, Jorge Luis Maria Ruiz, Marluce da Cunha Mantovani, Sérgio Paulo Bydlowski

**Affiliations:** 1 Organoid Development Team, Center of Innovation and Translational Medicine (CIMTRA), University of São Paulo School of Medicine, Sao Paulo 05360-130, Brazil; antonio_biomed@hotmail.com (A.C.R.-F.); marlucem@gmail.com (M.d.C.M.); 2 Lipids, Oxidation and Cell Biology Team, Laboratory of Immunology (LIM19), Heart Institute (InCor), University of São Paulo School of Medicine, Sao Paulo 05403-900, Brazil; d.levy@hc.fm.usp.br; 3 Life and Nature Science Institute, Federal University of Latin American Integration-UNILA, Foz de Iguaçú, PR 858570-901, Brazil; jorge.ruiz@unila.edu.br; 4 National Institute of Science and Technology in Regenerative Medicine (INCT-Regenera), CNPq, Rio de Janeiro 21941-902, Brazil

**Keywords:** hematopoiesis, hematopoietic stem cells, stem cell culture, 2D culture, 3D culture

## Abstract

Hematopoiesis is the main function of bone marrow. Human hematopoietic stem and progenitor cells reside in the bone marrow microenvironment, making it a hotspot for the development of hematopoietic diseases. Numerous alterations that correspond to disease progression have been identified in the bone marrow stem cell niche. Complex interactions between the bone marrow microenvironment and hematopoietic stem cells determine the balance between the proliferation, differentiation and homeostasis of the stem cell compartment. Changes in this tightly regulated network can provoke malignant transformation. However, our understanding of human hematopoiesis and the associated niche biology remains limited due to accessibility to human material and the limits of in vitro culture models. Traditional culture systems for human hematopoietic studies lack microenvironment niches, spatial marrow gradients, and dense cellularity, rendering them incapable of effectively translating marrow physiology ex vivo. This review will discuss the importance of 2D and 3D culture as a physiologically relevant system for understanding normal and abnormal hematopoiesis.

## 1. Introduction

**Figure 1:**
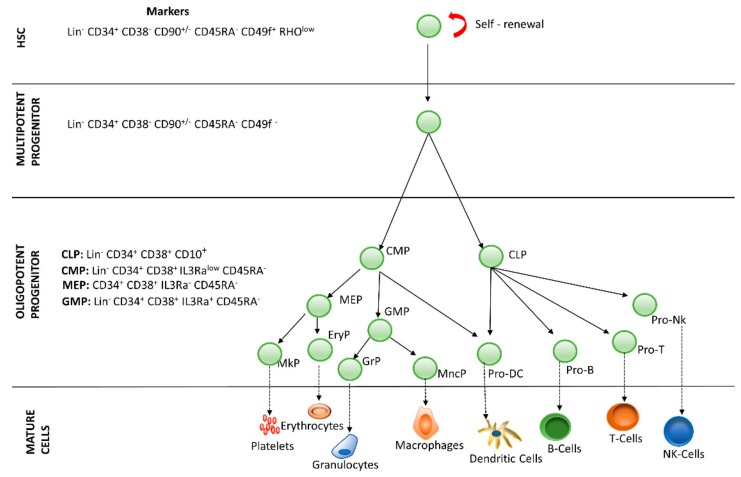
Hierarchy of human hematopoiesis. LT-HSC: Long Term-Hematopoietic Stem Cell; ST-HSC: Short Term-Hematopoietic Stem Cell; MPP: Multipotent Progenitor; OPP: Oligopotent Progenitor; LRP: Lineage Restricted Progenitor; MEC: Mature Effector Cell. The markers of the most important lineages are listed: Common Lymphoid Progenitor (CLP); Common Myeloid Progenitor (CMP); Megakaryocyte-Erythrocyte Progenitor (MEP); Granulocyte-Macrophage Progenitor (GMP). Restricted lineage progenitor cells: Megakaryocyte Progenitor (MkP); Erythrocytic Progenitor (EryP); Granulocytic Progenitor (GrP); Monocyte Progenitor (MncP); Dendritic Progenitor Cell (Pro DC); Progenitor Cell-T (Pro-T); Progenitor Cell-B (Pro-B); Progenitor Cell-Nk (Pro-Nk).

Blood is a connective tissue made up of approximately 34% cells and 66% plasma, transporting nutrients, gases and molecules in general to the whole body. Hematopoiesis is the process by which blood cells are formed, replenishing the blood system over the life of an individual. The hematopoietic process is a highly hierarchical phenomenon, in which hematopoietic stem cell (HSCs) differentiation and proliferation are of vital importance. Each cell within the hematopoietic hierarchy can be distinguished based on specific surface markers, which contain epitopes that are recognized by antibodies [1,2,3]. Figure 1 shows the main markers in human hematopoietic hierarchy. The hematopoietic process in humans starts in the yolk sac (mesoblastic phase). Then, it is transferred to the liver and spleen. Finally, the bone marrow becomes the main organ responsible for hematopoiesis. In the bone marrow, HSCs have the capacity of unlimited self-renewal, producing progeny that is the same as the original cell. They are generally in the G0 phase of the cell cycle and have the capacity to differentiate into specialized cells.

## 2. Hematopoietic Stem Cells

The medullary microenvironment participates in the quiescence, self-renewal, proliferation, maturation and apoptosis of HSCs and contains several cells (i.e., mesenchymal stem cells, endothelial cells, fibroblasts, osteoblasts, reticular cells, adipocytes). These cells are sources of cytokines, growth factors, glycoproteins and glycosaminoglycans, among other regulators. Different combinations of these molecules lead to the formation of specific microenvironments within the medullary cavity, known as niches [4]. Histologically defined microenvironments are subdivided into four regions: endosteal, subendosteal, central, and perisinusoidal. Granulocytes and monocytes are found in all regions of the bone marrow, whereas erythroblasts proliferate preferentially in the central region [5]. Concerning the dynamics of the lymphoid lineage, B lymphocyte precursors are found in the subendosteal region, gradually decreasing toward the central region, whereas mature B cells are found throughout the bone marrow [6]. HSCs are located in the endosteal region, also known as the osteoblastic niche, but studies suggest that HSCs may migrate to the perisinusoidal region or vascular niche and remain quiescent or differentiate depending on the needs of the organism [2,5,7,8,9]. In fact, studies with new markers for HSCs and niche cells, new image techniques, including labeling protocols, have shown that most HSCs reside adjacent to sinusoidal vessels, leading to the proposed existence of a perivascular niche for HSCs [10]. It is assumed that in the bone marrow, there are at least two different niches: the endosteal niche, which would harbor quiescent HSCs, and the perivascular niche, which would harbor cycling HSCs [11]. Although most studies have been done on non-humans, researchers suppose that the data reflect what happens in humans. It has previously been proposed that HSCs are maintained in the endosteal (osteoblastic) niche; however, the available evidence does not seem to support this model. Nevertheless, the endosteal niche seems to support the maintenance of a subset of lymphoid progenitors [10]. Approximately 80% of dividing and non-dividing HSCs have been described to be associated with sinusoidal vessels, with another 10% of HSCs being adjacent to arterioles, and almost another 10% in transition zone vessels [10]. A small percentage of HSCs are located in the endosteum. In fact, a quantitative model of cellular components that could define these microenvironments, and the preferential location of HSCs in the bone marrow are still lacking [12]. Obstacles to recognizing the HSCs in the bone marrow include the low frequencies at which HSCs are found in the bone marrow and the cellular complexity of the bone marrow microenvironment.

Cell signaling in the HSC niche is a complex process and passes through an extensive signaling network balancing self-renewal and differentiation [13]. This signaling involves several substances, such as growth factors and cytokines that are secreted by both the medullary stroma cells and hematopoietic stem cells and are important signaling factors for hematopoiesis, proliferation, and differentiation [14].

The Jak/STAT, Ras/Erk, and PI3K/Akt signaling pathways have been described as important inducers of erythropoiesis transduction by activation of the erythropoietin receptor, and these intracellular pathways are responsible for the survival, proliferation, and differentiation of normal erythropoietic progenitors [15].

Other intracellular signaling pathways that are important for the control of hematopoiesis have been described, such as the Notch, Wingless-type (Wnt), and Hedgehog pathways [16], which have been associated with self-renewal and maintenance of HSCs. Notch proteins are highly conserved receptors on the surface of the cell membrane that regulate the development of stem cells, and mutations in this receptor may cause leukemia [17] and breast cancer [18]. Activation of the Notch pathway is necessary to keep HSCs undifferentiated. This pathway is more active in HSCs and less active in differentiated cells. Inhibition of the Notch pathway potentiates the differentiation of HSCs and loss of the bone marrow reconstitution capacity of sub-lethally irradiated animals; thus, Notch has been used as a marker of undifferentiated HSCs [19]. Wnt protein regulates several phenomena during fetal development, and this protein has been related to the self-renewal of stem cells [20]. Hedgehog (Hh) protein has been described as regulating embryonic and adult stem cell activity. In mammals, three genes are known to be responsible for this protein—Sonic Hedgehog (SHh), Indian Hedgehog (IHh), and Desert Hedgehog (DHh) [21,22]. 

Soluble factors are also closely associated with the maintenance and regulation of the undifferentiated state of HSCs in the bone marrow of adults, in addition to regulating the proliferation and differentiation of this population. Stromal-cell-derived factor-1 (SDF-1/CXCL12) and its CXCR4 linker are activated to recruit endothelial progenitor cells (EPCs) and regulate HSCs [2,13,23]. Other soluble factors act to promote the maintenance of HSCs in their niche; for example, the stem cell factor (SCF/Kit-ligand) and its c-Kit receptor (CD117) are both required by HSCs for their maintenance. SCF is an important soluble cytokine for hematopoiesis, and THE c-Kit receptor is expressed on the HSCs surface; altered forms of this receptor have been associated with several types of cancer [24,25]. Thrombopoietin (TPO) and its MPL ligand are also important soluble factors necessary for the maintenance of HSCs in their niche. TPO is a primary physiological regulator responsible for the stimulation of platelet production, a primary dominant factor and megakaryocytopoiesis stimulator. In addition, recent in vitro studies have shown that TPO alone or in combination with growth factors, such as a c-Kit ligand, IL-3, or even Flt-3, stimulates the proliferation of hematopoietic progenitor cells [26,27]. Many other factors also modulate the function of HSCs but are not necessarily required, such as angiogenin, angiopoietin-1, G-CSF, IL-6, and TGFβ, among others [25].

## 3. Stem Cell Culture Methods

The complex microenvironment and cellular interactions are difficult to reproduce in vitro. Some improved techniques can help researchers mimic the bone marrow architecture for hematopoiesis studies and cell production in regenerative medicine applications.

Cell culture is the growth of cells from animal or plant in a favorable, artificial, and controlled environment. Today, cell culture is the basis of biology techniques and essential for regenerative medicine procedures [28]. These cells can originate directly from the tissue or after enzymatic or mechanically dissociated tissue or can be derived from an already established cell line. 

**Figure 2:**
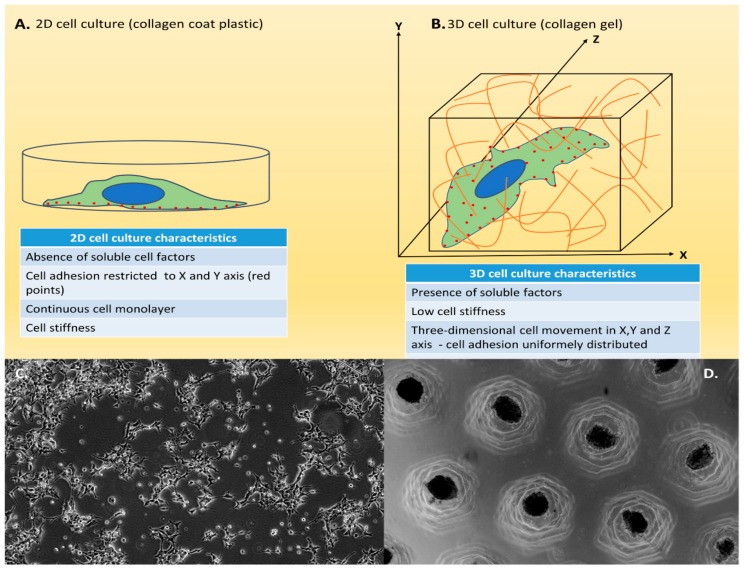
Two-dimensional (2D) and three-dimensional (3D) cell culture. (**A**) Schematic model of 2D cell culture. Standard model of 2D cell culture. Cells are cultured as a single layer in a culture flask. (**B**) Schematic model of 3D cell culture. 3D model of cell culture giving the notion of height, width and depth; cells are surrounded by the medium. (**C**) Example of 2D culture. HEK 293 cells in 2D cell culture; the adherence to a flat surface provides mechanical support for growth in monolayers. Scale bar 400 μm. (**D**) Example of 3D culture. HEK 293 cells 3D cell culture in agarose allowing the cells to grow or interact with their surroundings in all three dimensions. Scale bar 1000 μm.

Traditional cell culture, also known as 2D cell culture, is a very well-established method and easy-to-use culture model (Figure 2A,C). This technique depends on adherence to a flat surface, typically a culture flask, dish or polystyrene glass, to provide mechanical support for its growth in monolayers and access to nutrients and growth factors present in the culture media. Cells can also grow in suspension, as those derived from blood or bone marrow [29]. This method may present some advantages, such as low cost and performance of the functional assays, but this type of culture may also present some disadvantages, such as not mimicking the natural structures of the tissue and not being able to efficiently mimic cell–cell and cell–environment extracellular interactions [29].

Primary cell culture has been one of the greatest tools of cellular biology for evaluating cellular aspects such as chemoresistance, karyotypes, cellular parameters, metabolism and in vitro modeling of physiological and pathological models [30]. The isolated cells resemble their tissue of origin. Primary cell culture is complex and requires specific care, including storage, thawing-freezing procedures, or choice of enzymatic treatment, but the main problem is how to keep the primary cell in culture long enough to be established for experimental tests [31,32,33]. Primary culture can be representative of the cell types of the tissue from which they were isolated; although these cells are difficult to maintain, they best mimic a pathological condition or physiological function.

Immortalized cells or established cell lineages are cells that can grow in vitro indefinitely due to natural or induced transformation (e.g., embryonic stem cells or viral transformation) [34]. Immortalized cells have several advantages over primary cells, such as profitability, easy manipulation, unlimited supply of material, and lack of ethical concerns associated with the use of animal and human tissues [35]. Immortalized cells also provide a uniform and homogeneous population of cells [36]. Immortalized cell lines emerged as a solution to some problems that appeared with primary cell cultures, such as misidentification of the cell line used and genotypic and phenotypic instability. Care should also be taken to avoid the use of aged cell lines that do not maintain the original physiological characteristics. The immortalized strains constitute a simple and representative model system for functional studies and therapeutic targets. As each cell line may have unique characteristics, specific studies should take these characteristics into account [37].

The co-culture technique is used for numerous applications, including the study of natural or synthetic interactions between distinct populations of cells. Co-culture methods are of great importance in research, as they are used to observe cell–cell interactions, how cells are organized, how they participate in the development of diseases such as cancers, in which different types of cells are involved, including the microenvironment [38].

Both self-renewal and differentiation are required abilities in any cell of the hematopoietic lineage [39]. However, the expansion of these cells in vitro has been challenging for the scientific community, as regulation of these cells depends on several mechanisms of intercellular communication resulting from the secretion of local- and systemic-acting factors [40]. HSCs, which can also be obtained and expanded from umbilical cord blood, should respond to and integrate events in their microenvironment to ensure sustained production of all hematopoietic lineages [41]. 

Although HSCs have been extensively analyzed and characterized, their ex vivo expansion remains a problem [42,43,44]. It has been shown that the cell culture of HSCs is viable for at least 169 days but very much depends on the quality of the HSCs. There are currently several approaches to ex vivo expansion. Most first require the isolation of CD34^+^ or CD133^+^ cells from frozen or fresh hematopoietic tissue and incubation in culture medium supplemented with cytokines, granulocyte colony stimulating factor (G-CSF), stem cell factor (SCF), and thrombopoietin (TPO) [45]. 

Therefore, the cell culture of HSCs must take into account the microenvironment in which these cells are inserted so that it can reproduce the whole framework of the hematopoietic structure. The hematopoietic niche contains several types of cells, and mimicking this microenvironment in vitro requires a stromal layer, which controls multiple cellular parameters, including quiescence, self-renewal, differentiation, apoptosis and migration. Under the artificial culture conditions, HSCs undergo differentiation and apoptosis [46]. A support layer is required so HSCs can survive and proliferate.

Co-culture of HSCs is one of the most frequently used models for understanding how the highly specific bone marrow niche interacts ex vivo with hematopoietic cells, promoting their differentiation and expansion. The technique can focus on the importance of non-contact culture systems on the successful maintenance of hematopoietic cells [47] and on the use of the stroma as a cell layer to provide support for HSC culturing, as mentioned above. Several studies have shown that contact between HSCs and stromal cells is important for maintaining HSC function [48,49]. The stromal cells from bone marrow include osteoblasts, macrophages, endothelial cells, and mesenchymal cells [50,51]. Mesenchymal cells are multipotent progenitor cells that can differentiate into mesenchymal cells, such as osteoblasts, adipocytes, and chondrocytes, and also have the potential for differentiation into cells such as neurons and lung cells [52]. These multipotent cells have interactions with hematopoietic cells forming the framework of the HSC niche, supporting the development of hematopoiesis and acting as immunological regulators [53,54]. In addition, recent studies have indicated that other molecules, such as N6-methyladenosine (M6A), by modulating the expression of a group of YTHDF2 genes at the mRNA level, are important regulators of HSC self-renewal. Some authors have suggested that although extensive efforts have led to multiple methods for in vivo expansion of HSCs, it is not possible for some single molecules or pathways to be manipulated simultaneously due to a large number of essential targets for self-renewal of stem cells [55].

In this regard, there are still important challenges to overcome, including the development of more efficient methods for the maintenance of HSCs in vitro, and methods of ex vivo expansion for therapeutic development in regenerative medicine; then, it will be possible to have a better understanding of the hematopoietic niche and its intrinsic and extrinsic regulators from a physiological and pathophysiologic point of view. The ability to genetically reprogram HSCs for clinical therapeutic use [56] still needs to be improved.

## 4. 3D Hematopoietic Stem Cell Culture

Cell culture in 2D format is simple and provides excellent material for studying homogeneous populations [57]. However, it does not consider several other important parameters of cell physiology, such as cell–cell communication or communication between the cell and microenvironment or adjacent molecules [58]. Therefore, the major problem with 2D cell culture is its limits [59]. One of the main criticisms of this study format is that animal physiology cannot be mimicked using only one layer of cells, which certainly does not correspond to the original tissue considering the complexity of the cellular microenvironment in the tissue of origin [60]. 

As an alternative to the technical limitations of 2D cell culture, 3D cell culture allows a better simulation of the in vivo structural complexity, replicating several characteristics present in tissues (Figure 2B,D), not only the interaction of cells with their microenvironment, but also morphology, differentiation, polarity, proliferation rate, gene expression profiles, and cell heterogeneity [61,62,63,64]. In fact, 3D culture has proven to be a realer model for translating research results into in vivo applications.

Current 3D cell culture models and methods include spheroids, organoids, microcarrier cultures, organs-on-chips, and 3D bioprinting [65,66]. Organotypic explant culture methods are used mostly when a technical/specific requirement of the tissue is to be studied and mimicked [67]. However, although the complexity of the 3D system is evident, some criteria must be considered and cannot be disregarded, such as the choice of material for the scaffold and hydrogels and cell type and culture methods, which vary considerably according to the tissue studied [66]. 

Organoid culture is most commonly used to describe constructs derived from pluripotent stem cells (embryonic or induced cells) or adult stem cells from various organs, including the hematopoietic tissue [68]. Organoid culture is classified as either tissue organoids (i.e., organotypic) or stem cells depending on how the cell layers are formed [69]. Organotypic tissue refers to free stromal cells without parenchyma, and its application is mainly with epithelial cells because of their high intrinsic ability to self-organize. With this method, it is possible to study HSCs, and Christopher et al. [70] were able to produce mature T cells from stem cells and hematopoietic progenitors in a thymus organoid culture. 

Spheroid culture models simulate the microenvironment conditions of a living cell. Compared to the classic 2D model, the spheroid culture model emphasizes the interactions between cells and their relationship to the extracellular matrix (ECM) [71,72]. Tissues are not composed of a homogeneous population of cells; they are complex structures formed by several different components, with an intricate relationship such as vessels, nerves, and stroma, which should be considered in any kind of tissue engineering. This complex interaction between cells and the extracellular compartment requires a 3D environment to best represent these interactions [73,74]. 

Spheroid cell culture can be used in scaffold and scaffold-free models. The scaffold methods comprise hydrogel support, decellularized extracellular matrix, and resistant polymeric material support. Hydrogel consists of polymeric groups with a water-swollen hydrophilic structure [75]. Depending on their nature, these compounds are classified into different categories, including ECM protein–containing hydrogels (ECMPs), natural hydrogels, or synthetic hydrogels, each one with its own properties [76,77]. The decellularization technique consists of removing cells from the native tissue by chemical treatment, preserving the ECM. The tissue can then be replenished with cultured cells and grafted in vivo or used for ex vivo cell differentiation. In contrast to artificial scaffolds, decellularized scaffolds preserve the vascular structure, tridimensional niches, and chemical composition of bone marrow. Extracellular matrix components are directly related to several important factors for cell survival support, including cell behavior, signaling for survival, and proliferation, among others. Resistant polymeric material supports are structures similar to fibers or sponges. Cells maintained in this type of support exhibit a physiological behavior close to that of native tissue (such as those of cartilaginous tissue). Regarding the polymeric support, the most commonly used material is polystyrene, but biodegradable supports such as polycaprolactone have also been employed. 

The development of a bioprinting system for the bone marrow microenvironment is important since it cannot be mimicked by methods such as organoids. Advanced 3D bone marrow models could serve for several different studies, including hematopoietic regulation, BM, MSCs, HSC interaction and expansion, interactions of hematological cancer cells, and evolution of several hematological diseases. In addition, these models could be used as a platform for expanding HSCs for transplantation. However, it is difficult to obtain a reliable BM model because of several critical technical challenges, from currently available 3D printing techniques to the possibility of precisely mimicking the different BM niches [78].

Relatively few studies have been done using hematopoietic stem cells in these methods. Decellularization of cartilaginous tissues has been tested for ex vivo culturing of hematopoietic cells [79]. A co-culture model of hematopoietic stem/progenitor cell (HSPC) spheroids using polydimethylsiloxane (PDMS) has shown that the effectiveness of three-dimensional culture for HSPC expansion for clinical use is still a strategy that needs further improvement [80]. 

A three-dimensional collagen-based culture using HSPC, bone-marrow-derived MSCs or the umbilical cord (UC) to mimic the main compartments of the bone marrow hematopoietic niche has been proposed [81]. Data analysis generated the following compartments: (I) HSPC in suspension above collagen and (II) migratory HSPC in collagen fiber matrix. The different sites were representative of the distinct microenvironments that make up the bone marrow and have a significant impact on the fate of HSPC. The authors suggested that this 3D culture system using collagen and BM-MSC allowed HSPC expansion and provided a potential platform for advanced study of niches and hematopoiesis and their regulatory mechanisms. 

A bone marrow composed of two compartments, solid and liquid, that act harmoniously, has been proposed [82]. A bone marrow structure was created using a macroporous PEG hydrogel that resembled the macroporous 3D architecture of the trabecular bone, site of the red bone marrow, and, therefore, where the HSPC niches are located. This bone marrow analog was found to be suitable for HSPC culture and for enhancing HSPC expansion compared to conventional 2D cell culture. The developed model of a perfused 3D bone marrow analogue mimicked the HSCs niche under steady-state or activated cell conditions that favor the maintenance or differentiation, respectively, of HSCs and allowed drug testing [82] It was concluded that the system reflected the behavior of HSPC in the niche under physiological conditions.

Some studies have shown that the interaction of bone marrow stromal cells with leukemic cells increases the resistance of these leukemic cells. Numerous scaffolds have been created to provide a minimal structure based on the 3D leukemic microenvironment [73]. As 3D culture could result in resistance to drugs, it can be a good screening tool for drug evaluation prior to the administration of chemotherapic drugs [74,83]. A classic example of scaffolds used to recreate the leukemic niche is polycaprolactone, which is an aliphatic and biodegradable polyester with good biocompatibility [84,85].

Recently, a scaffold of degradable zwitterionic hydrogel was tested for human HSPC expansion [86]. A 73-fold increase of long-term hematopoietic stem cells was observed (LT-HSC). The viscoelasticity and smoothness of the highly hydrated zwitterionic hydrogels seems to be important for the creation of cell niches, by their unique mechanical and antifouling properties. 

The organs-on-chips technique was developed to study the mechanical and physiological response of a tissue, combining concepts of tissue engineering and microfluidics [87]. A bioelectronic device based on a conductive polymer scaffold was integrated with an electrochemical transistor configuration that allows 3D cell growth and the real-time monitoring of cell adhesion and growth. 

This technique consists of manipulating small amounts (10^−9^ to 10^−18^ L) of fluids using small openings with micrometer dimensions. This methodology has been widely reported because it offers several advantages, such as using small amounts of samples and reagents, performing separations and detections with high resolution, and a short time for analysis [61]. Such techniques allow the precise control of fluids and particles in a given cell culture. This culture method enables the control of such nanoliter-scale fluids as described above and further enables and facilitates the simultaneous manipulation of cultured cells from a single cell [62]. Through precise manipulation of the components of the microfluidic culture medium, it is possible to transport nutrients, hormones, and oxygen growth factors to facilitate homeostasis and recreate mechanical signals that are absent in traditional culture [88].

This cell culture system offers several advantages for basic research applications, including the precision of micromanufacturing, which allows the presentation of a controllable and reproducible microenvironment [64]. Another advantage of this model is that it provides complete control over the conditions of the cell culture, including dynamic cell control, nutrient addition, removal of metabolites, stimulation with drugs and proteins, and simultaneous image and chip format [62,89,90]. 

The organs-on-chips technique was described in cancer studies (cancer-on-chip), where it can replicate the microenvironment to achieve robust and reliable results [91,92]. However, up to now, it has been difficult to culture HSCs in an organs-on-chips model. A device with a central cavity that successfully mimics the bone and bone marrow has been described. This device is made from polydimethylsiloxane (PDMS) with bone inducers inserted. The central chamber of this device is composed of porous PDMS membranes, and cytokines are added in the microfluids [93]. Although several methods have been used, results using HSCs are generally poor [94].

## 5. Final Remarks

Bone marrow HSCs are the stem cell most used in medical practice. Finding effective ways to mimic the bone marrow HSCs and microenvironment, the hematopoietic niche, in vitro is a challenge. In addition to HSCs, several other bone marrow cell types, including megakaryocytes, macrophages, monocytes and endothelial cells, directly or indirectly regulate HSCs and niche function. Two-dimensional cell cultures have been widely used due to their low maintenance cost and easy learning. However, 2D culture has several limitations. As a result, 3D cell culture techniques have been developed. Although the current cost is relatively high, the fact that a 3D culture has characteristics much closer to the tissue being studied is a great advantage. There are several methods currently employed or in development, such as spheroids, organoids, 3D bioprinting and organs-on-chip, and these are facing the challenge of culturing HSCs while maintaining their properties. This achievement will allow ex vivo HSC expansion, bringing new medical perspectives for HSCs transplantation, drug testing, and personalized treatment.
